# The Early History of Veterinary Medicine in the United States

**Published:** 1884-01

**Authors:** R. Jennings


### Art. IV.—THE EARLY HISTORY OF VETERINARY MEDICINE IN THE UNITED STATES.

BY R. JENNINGS, V.S.

*Continued from page* 49, *Vol.* 4.

IN the year 1865 the younger members of the veterinary profession, becoming dissatisfied with the monotony from the long continued efforts and slow progress made by the Philadelphia Veterinary College, conceived the idea that the interests of the veterinary profession would best be promoted by inaugurating a new movement in the interest of veterinary science. The subject was fully ventilated at a meeting of the Veterinary Association. When, on motion of James McCoart, it was resolved, that the interests of the veterinary profession would be advanced by obtaining a charter for a new college with a new faculty, to be known as the Pennsylvania College of Veterinary Surgeons, under the exclusive control of the veterinary profession. This, it was believed, would give the movement a new impulse, improve its vitality and insure its success. Measures were at once taken to secure the passage of a new act of incorporation. A petition was drawn and presented to the Legislature of Pennsylvania and was duly granted April 11th, 1866.

On receipt of the charter the college was organized, and officered as follows:

Isaiah Michner, V.S., Professor of Theory and Practice of Medicine; Robert Jennings, V.S., Professor of Pathology and Surgery; Michael W. Birch, V.S., Professor of Materia-Medica and Pharmacy; James McCoart, V.S., Professor of Anatomy and Physiology.

Clinical Lecturers—T. B. Rainer, V.S.; R. Jennings, V.S.; Isaiah Michner, V.S.; T. J. Corbin.

John Berry, V.S., Dean.

The prompt action of the Association in selecting a faculty and making arrangements for putting the new institution in immediate working order induced the Directors of the Veterinary College of Philadelphia to close its doors at the end of the session for 1865-6. Thus ended the career, March 3d, 1866, of the first veterinary college in America. The new institution fitted up a building at the corner of Columbia Avenue and Sixth Street, adapted to its wants. All went well for about two years. The continued drain upon the purse strings of the members of the association soon had its effect upon their ardor. One by one withdrew their contributions or assessments made upon them, until the tax upon the few that remained firm became a heavy burden. There were no outside friends to extend a helping hand; all had been ignored. Two years later the Pennsylvania College of Veterinary Surgeons closed its doors, after the brief but uneventful career of four years.

### The United States Veterinary Medical Association.

The origin of this association having been questioned, I wish to put upon record such evidence as will forever settle the matter, and place the credit where it justly belongs. The following letters I put in evidence:

Boston, June 20th, 1859.

*Friend Jennings:*

Your letter of May 20th, 1859, duly received. * * * * Now as to the "National Association:" I fully agree with you, and both myself and friends here will hold ourselves in readiness to attend a meeting in New York city for the purpose of such an organization at any time that will best suit the convenience of parties desirous to unite with us in the cause.

Perhaps you had better consult with your friends on the subject and inform us of their views, either Copeman or myself, and we will attend to it in our parts of this union. "Union," did I say? Yes, that is just what we want. You may attend to this matter at your early convenience, so as to enable us to make our arrangements to meet in "convention." I remain, with great respect,

Yours truly

CHAS. M. WOOD,

---

Boston, Mass, May 25th, 1860.

*Dr. R. Jennings:*

Dear Sir—Yours of the 22d ult. came duly to hand. * * * In regard to the National Veterinary Medical Association I am afraid that you could not get enough "vets" together to make it an object. Jealousies and prejudices are very strong among the members of the craft, mainly graduates from the other side of the water look with supreme contempt on all who will not endorse the creed or dogmatical diction of the autocratic schools. Then again, you are aware that you, as well as myself, have enemies within and around our fields of practice, that from mere feelings of jealousy they will not give us countenance nor support. Still if you deem it proper to form a N. V. M. A. you may count on my assistance and support. Accept my best respects, and have the goodness to tender the same to your associate, Dr. Bowler.

Yours truly,

G. H. DADD.

---

By the above letters it will be seen that I had in view several years previous the establishment of a National Veterinary Medical Association, and its being endorsed by Dr. Chas. M. Wood and Geo. H. Dadd, of Boston, Mass., is significant of the fact.

PHILADELPHIA, March 3d, 1863.

At a meeting of veterinary surgeons held this evening at the Wetherill House, Dr. R. Jennings suggested the propriety of calling a national convention of veterinary surgeons for the purpose of advancing the standard of veterinary medicine and surgery in the United States, seconded by Dr. J. C. Higgins, of New Jersey, and on motion unanimously adopted; Dr. W. A. Wisdom, of Delaware, in the chair; Dr. R. Jennings, was appointed Secretary, when it was

*Resolved*, That the formation of a National Veterinary Association not having been previously anticipated, the President be and he is hereby authorized to call, at an early day, a meeting of the veterinary profession of the United States for the purpose of permanent organization. On motion,

*Resolved,* That a national convention of veterinary surgeons be called to meet in the city of New York or Philadelphia some time during the coming summer, the time and place to be decided at the next meeting.

*Resolved,* That the Secretary be, and he is hereby requested to communicate with members of the profession in the several States, requesting their attendance at the convention when called. Veterinary surgeons present, Wm. A. Wisdom, Del.; Jacob Dilts, J. C. Higgins, A. Phillips, New Jersey; R. J. Wilson, England; R. Jennings, R. McClure, E. H. Palmer, T. B. Rainor, I. Michener, Pa.; G. W. Bowler, Ohio; W. J. McCoun, New York.

At a subsequent meeting held April 28th, 1863, the following letters were read.

NEW YORK, March 28th, 1863.
No. 407 Fourth Ave.

DEAR SIR—I observed in a recent number of Wilkes Spirit of the Times that at a meeting of veterinary surgeons held in Philadelphia, it was proposed to form a "National Veterinary Association;" that resolutions to try and effect that purpose had been adopted; that Mr. Wisdom and yourself were requested to correspond, "with such members of the profession as they may be acquainted, soliciting their aid in support of the proposed measure, and their attendance at the convention when held." The wording of the resolutions would seem to debar all who were not veterinary surgeons from taking any part or interest in the formation of the association. Although not a veterinary surgeon, I am deeply interested in all that pertains to that much (in this country) neglected and important science. I have been for the last sixteen or eighteen years trying to elevate that branch of comparative anatomy to a more eminent position than it occupies at present. It will afford me much pleasure if I can in any manner assist you or your associates in perfecting the object proposed. That such association is much needed there can be no doubt, that it will not only be beneficial to the profession, but to the country at large, and the government in particular. I think with you, that the time has arrived for veterinary surgeons and others practicing or interested in the art to claim for themselves a status to which they are, when properly educated, entitled. It is deeply to be regretted that the government, while acknowledging by letter to me the importance of veterinary science, has taken no steps towards forming an army veterinary department, although frequently urged so to do by myself, and I believe by many others.

I think New York would be the most appropriate place for the first meeting, and that Agassiz would be the most suitable person to deliver the introductory lecture. I believe my suggestions would meet the views of our mutual friend Copeman. Hoping that your long cherished desire may be speedily accomplished, I remain

Very respectfully,

R. Jennings, Esq., JOHN BUSTEED,

Veterinary Surgeon.

---

Utica, N. Y. April 4th, 1863.

Dear Sir—In acknowledging the receipt of your favor of yesterday, I beg to present for the early consideration of yourself and associates the propriety of making some alteration in the name or title of the "National Veterinary Association." You too well know the bitter enmity, strong prejudice, and mean, petty jealousy, now existing among veterinary surgeons in the United States. Now it occurs that a plan can be devised by which all opposition may be neutralized if not entirely overcome. The title, "National Veterinary Association" in my humble opinion, is another specimen of "lofty tumbling," of which we have had already too much; besides, this title is a "misnomer." I know the delicate nature of this matter and shall content myself with a simple statement of facts. If the gentlemen would consent to a change of title, say to the National Society for the Advancement of Veterinary Science, or Knowledge, a door would be opened for the admission of all competent and honorable workmen, all true patrons of the art, as well as those who admire knowledge simply for its real worth and power. By adopting such a name all party-feeling and strife may be prevented. Doctors know the value of this term. Proper rules would effectually exclude all unworthy persons. "I, for one," am anxious to see "free trade" principles adopted. The number of veterinary surgeons, "graduates," of European colleges are but a mere fraction of those practicing under that title in our cities and towns, and to be candid I must admit that some of these "have made vets," by years of patient study, close and untiring observation, added to a long and extensive experience, in many respects are better "qualified to practice," than some of the two session (graduates) from "abroad." Let a liberal code be adopted that will bring together a large portion of the "working class" of our profession. Our motto should be *qui docet discit*, "He who teaches others informs himself." By all means hold the first meeting in New York city. Before preparing any remarks for the press, I should be pleased to get the views of yourself and colleagues upon the "amendment" to title herein advocated. Trusting you will favor me by an early notice, I remain,

Truly yours,

A. S. COPEMAN.

---

NEW YORK, May 4th, 1863.

DEAR SIR—In answer to your letter of May 2d, for your kindness in appointing me as one of a committee for forming a National Association to advance veterinary science in this country where it is greatly needed, I am obliged to decline the honor, as my health is very much impaired by close attention to the duties of my profession, and I think of going to Europe with the hope of recuperating myself by a little rest. I must convey to you my best wishes for your success in so praiseworthy an undertaking even at this late date. I remain,

Yours truly,

C. C. GRICE, V.S.

---

OYSTER BAY, L. I., May 13th, 1863.

FRIEND JENNINGS—I have just returned from New York. I called upon Mr. Chas. Stetson, of the Astor House, and communicated to him our proposed plans for the National Convention to be held on the 9th of June. He is a great admirer of the profession, and is willing to lend us all assistance that may lay in his power. He offered me gratuitously the use of a large parlor to accommodate 75 or 100 gentlemen. I now submit his kind offer to you. I called upon Dr. Busteed. He thinks well of this. To give the thing tone and respectability we must go to a respectable place, and the Astor House has a wide-spread reputation as such.

Yours truly,

WM. T. McCOUN.

---

BOSTON, May 14th, 1863.

*Dr. R. Jennings:*

DEAR SIR—Your letter of May 2d has come to hand. * * * * I am aware of the necessity of the coöperation of all the veterinary practitioners to give strength and efficiency to the order. But permit me to say that I beg to be informed as to what are the qualifications required in such as may form the proposed convention. There are many persons who have taken up the practice of veterinary medicine and surgery who have had no proper instructions in those subjects and are entirely ignorant of the principles on which they are founded. They have assumed the title and duties of professional men only for the name and living which may be derived from it. No man who knows me, but is acquainted with the earnestness of my efforts to extend veterinary science, and I have labored to show the loss and injury which always attends the ministrations of quack practitioners. The quacks of our profession have never had any respite. Whenever I come in contact with them, or was summoned to arrest the progress of the evils they had inflicted, I have stood almost alone in this city espousing the cause of legitimate science, and my efforts have not been unavailing. Our people have discovered that veterinary science requires learning as well as medical science, and that new discovery has been confined by the fatality which has attended the operations of the unlearned. In a letter a few days since from my esteemed friend, Copeman, he informed me that he intends to be present at the convention. And at a meeting of our "vets." here last evening it was voted for several to come, but we must wait your reply to this for information asked for. I now remain, friend Jennings,

Yours truly, and a laborer in the cause,

CHAS. M. WOOD.

---

New York, May 13th, 1863.
98 East 13th Street.

*R. Jennings, Esq.:*

Sir—I am in receipt of your favor of the 4th inst., and would most respectfully decline the honor you would confer upon me by appointing me upon a committee for the diffusion and advancement of veterinary knowledge. My business and other engagements are such at this particular time that it precludes me from taking any active part in this very meritorious work. Hoping my refusing to act may not seriously incommode you in your undertaking, I am yours,

Very respectfully,

W. W. A. M. LOCKHART, V.S.

---

College Place, Brooklyn, N. Y.

*R. Jennings. Esq.:*

Dear Sir—I have just received your letter, the object of which I highly approve, but as I have been out of practice some years and am much engaged in other business, I am compelled to decline serving on the committee, at the same time I would suggest my adopted son, Alfred Large, veterinary surgeon and member of the Royal College of Veterinary Surgeons of London, in my place. If this alteration meets the views of yourself and friends, write me by return of post and he will attend to the matter without delay.

Yours very respectfully,

R. M. CURTIS.

---

New York, May 15th. 1863.
179 Lexington Avenue.

Dear Sir—I received yours of the 14th inst., appointing me to confer with the members of the veterinary profession in this city in making arrangements for a meeting to be held here the 9th of June next. My acquaintance with my professional brethren is exceedingly limited, having been myself but a short time in America. I will, however, be most happy to confer with them upon any subject pertaining to the advancement of veterinary education and science. Dear sir,

Yours ob't,

A. LIAUTARD.

R. JENNINGS, ESQ.

---

After the reading of the above correspondence the following preamble and resolutions were read and adopted unanimously:

*Whereas*, Veterinary science in this country has been kept in comparative obscurity in consequence of its practice having been confined mainly to the hands of men uneducated in the anatomical and pathological relations of the various diseases to which our domestic animals are subject, as well also as to therapeutic action of the remedies used in combating disease. This deplorable condition of the veterinary profession has been the means of excluding the qualified practitioner from the army in the United States. The losses to the national government in consequence from the purchase of large numbers of horses unfit for the duties required of them, having amounted to millions of dollars, therefore,

*Resolved*, That the friends of veterinary science favorable to forming a National Veterinary Association for the advancement and diffusion of veterinary knowledge meet in convention in the city of New York on Tuesday, June 9th, 1863.

*Resolved*, That veterinary surgeons in all parts of the United States, and all persons favorable to such an organization be invited to attend the convention.

The Secretary offered the following, which was, on motion, accepted:

*Committee of Arrangements.*—New York city—John Busteed, M.D., W. T. McCoun, V.S., A. Liautard, V.S., Chas. Pilgrim, V.S., C. C. Grice, V.S., Robt. Curtis, V.S., and George Wilkes, Esq.

NEW YORK, June 9th, 1863.
Astor House.

Dr. W. A. Wisdom, of Delaware, called the convention to order, Dr. R. Jennings, of New Jersey, acting Recording Secretary.

The minutes of the two previous meetings, held in the city of Philadelphia, were read and accepted, (but were not recorded in the minute book of the United States Veterinary Medical Association.)

Dr. C. M. Wood, of Boston, moved that Dr. J. Busteed, of New York, be appointed Chairman of the meeting. Carried.

Delegates present: John Arnold, J. K. Quickfall, England; John Busteed, M.D., Louis Brandt, John F. Budd, C. Burden, C. H. Birney, W. H. Banister, A. S. Copeman, R. H. Curtis, C. C. Grice, A. Large, A. Liautard, W. T. McCoun, J. Mulligan, E. Nostrandt. New York; J. H. Budd, J. Dilts, T. Cooper, J. C. Higgins, Humphrey Sill. R. Jennings, W. R. Mankin, A. Phillips, J. Phillips, New Jersey; J. C. Essenwein, R. McClure, I. Michener, Geo. Miller, E. H. Palmer, Pennsylvania; W. Saunders, J. H. Stickney, E. F. Thayer, C. M. Wood, R. Wood, Massachusetts, W. A. Wisdom, Delaware; G. W. Bowler, Ohio; E. F. Ripley, Maine.

New York, June 10th, 1863.
Astor House.

This morning the convention assembled at 9 o'clock. Dr. J. Busteed in the chair.

Minutes of the previous meeting read and approved.

The Committee on Constitution, made their report, submitting a code of laws, which were amended and then adopted for the future government of the Association.

The following officers were elected to serve one year:

President—J. H. Stickney, Mass.

Vice-Presidents, R. H. Curtis, N. Y., W. Saunders, Mass., E. F. Ripley. Maine, R. Jennings, N. J., W. A. Wisdom, Del., G. W. Bowler, Ohio, R, McClure, Pa.

Recording Secretary—A. Liautard, New York city.

Corresponding Secretaries—W. J. McCoun, N. Y.; I. Michener, Pa.; R. Wood, Mass.; J. C. Watton, N. J.

Treasurer—A. S. Copeman.

Censors—A. Large, N. Y.; E. F. Thayer, C. M. Wood, Mass.; J. Dilts, N. J.; E. H. Palmer, J. C. Essenwein, Pa.

After considerable discussion the name "United States Veterinary Medical Association," was adopted, by which the organization will be known. Dr. R. Jennings exhibited the ecraseur for castrating horses, a French invention, introduced in the United States by him for that purpose in the year 1852, but it was not favorably received by stock owners. He explained its advantages and working, but it was not at that time appreciated by the members of the convention. Several interesting papers were read by A. S. Copeman, C. M. Wood and others, after which the convention adjourned.

